# AMR surveillance in Canada: Insights from the 2025 Priority Pathogen List

**DOI:** 10.1371/journal.pone.0341133

**Published:** 2026-02-18

**Authors:** Kahina Abdesselam, Pia K. Muchaal, Aanchal Mishra, Raymond-Jonas Ngendabanka, Kanchana Amaratunga, Sakhi Mittal, Wallis Rudnick, Anna-Louise Crago, Robyn Mitchell, Stephanie Alexandre, Tanya Lary

**Affiliations:** 1 Antimicrobial Resistance Task Force, Public Health Agency of Canada, Ontario Canada; 2 AMRNET, National Microbiology Laboratory Branch, Public Health Agency of Canada, Ontario Canada; Hawassa University College of Medicine and Health Sciences, ETHIOPIA

## Abstract

**Background:**

Antimicrobial resistance (AMR) remains a growing threat to public health and healthcare systems. In 2015, Canada published its first AMR Priority Pathogen List. In 2025, an updated list was developed to reflect evolving resistance trends, emerging pathogens, and advances in national surveillance capacity.

**Objective:**

To conduct a qualitative analysis of the 2025 AMR prioritization results to evaluate the strengths and limitations of current Canadian AMR surveillance systems in detecting and monitoring high-risk pathogens.

**Methodology:**

As described in a companion paper, 29 AMR pathogens were prioritized using a multi-criteria decision analysis (MCDA) framework, informed by Canadian data from 2017 to 2022. Pathogens were assessed across nine weighted criteria, including incidence, treatability, and a newly integrated health equity dimension. Criteria weights were derived from expert consensus, and sensitivity analyses confirmed ranking stability. This paper presents a qualitative analysis examining how well these pathogens are captured by existing national surveillance systems and highlights data integration gaps.

**Results:**

The 2025 prioritization identified significant shifts in Canada’s AMR landscape, including the emergence of *Candida auris*, drug-resistant *Neisseria gonorrhoeae*, and *Mycoplasma genitalium* as high-priority threats. Canada’s approach aligns closely with international frameworks (e.g., WHO, CDC), enhancing its global relevance. AMRNet, a national laboratory-based AMR surveillance platform, has the capacity to eventually capture 90% of prioritized pathogens, and over half of pathogens are currently covered by enhanced national systems that integrate laboratory and epidemiologic data. A quadrant-based assessment revealed strong coverage for several pathogens, but persistent gaps remain, particularly for those lacking routine diagnostics. Over one-third of prioritized pathogens disproportionately affect marginalized populations, underscoring the importance of integrating disaggregated sociodemographic data into surveillance.

**Conclusion:**

Canada has made meaningful progress toward integrated, equity-informed AMR surveillance. Continued investment and collaboration are essential to close persistent data gaps, improve responsiveness, and support targeted public health action**.**

## Introduction

Antimicrobial resistance (AMR) is widely recognized as a silent and escalating pandemic [1]. When antimicrobials stop working, it becomes harder to treat infections, putting lives at risk and undoing years of progress in treating infectious diseases [1–3]. In response, Canada has made AMR a national public health priority, reflected in the 2023 Pan-Canadian Action Plan on AMR (PCAP) [4], which outlines a coordinated One Health approach to leadership, surveillance, stewardship, and research and innovation. Canada’s global commitments, including alignment with the WHO Global Action Plan [5], the UN Sustainable Development Goals [6], and the Global Health Security Agenda [7], further underscore the urgency of addressing AMR as both a domestic and transnational threat [8].

To support a more coordinated national response, Canada established the Canadian Antimicrobial Resistance Surveillance System (CARSS) in 2014 [9,10]. CARSS has since served as the central platform for integrating and reporting on AMR and antimicrobial use (AMU) surveillance data collected across the Public Health Agency of Canada (PHAC), and for identifying data gaps and emerging trends to guide public health action [9–11].

The 2025 AMR Priority Pathogen Threats exercise [12] built on this foundation, offering updated evidence to inform not only surveillance, but also potentially research, stewardship, and infection prevention and control practices. Through a multi-criteria decision analysis framework, 29 pathogens were ranked according to their public health threat level using nine weighted criteria, including incidence, trend, mode of transmission, case fatality ratio, morbidity, treatability, detection, health equity and preventability. While the exercise was designed to guide future action, it also provided a valuable opportunity to assess the current strengths and limitations of Canada’s AMR surveillance systems.

This paper explores ongoing work to enhance AMR surveillance in Canada. It examines equity considerations and emerging solutions, such as integration of syndromic and pathogen-specific surveillance, expansion and tailoring of national surveillance programs including Antimicrobial Resistance Network (AMRNet) [13], Canadian Nosocomial Infection Surveillance Program (CNISP) [14], and Enhanced Surveillance of Antimicrobial Resistant Gonorrhea (ESAG) [15], and the need for molecular diagnostics and equity-informed data collection at the national level. Unlike the prioritization exercise, which applied a structured, criteria-based ranking of pathogens, this analysis draws on qualitative insights from the exercise and working group to surface cross-cutting challenges that constrain surveillance effectiveness across jurisdictions. The discussion does not evaluate individual program mandates or prescribe policy solutions. Instead, it offers high-level guidance to support strategic alignment and stimulate ongoing reflection within and across surveillance programs. By leveraging insights from the 2025 prioritization exercise, Canada is positioned to advance a more responsive, inclusive, and adaptive surveillance infrastructure capable of meeting the evolving threat of AMR.

## Methods

This study builds on the methodology and findings of *Canada’s 2025 AMR Priority Pathogens: Evidence-Based Ranking and Public Health Implications*, led by the Public Health Agency of Canada (PHAC) [12]. The primary objective of this follow-up analysis is to assess how the 2025 prioritization results can inform improvements to AMR surveillance infrastructure in Canada. Specifically, this qualitative analysis:

1. Compares the 2025 AMR Priority Pathogen List to the 2015 list to evaluate changes in methodology, pathogen inclusion, and tier rankings [16].
2. Assess how well the 29 prioritized pathogens are captured by existing national and provincial/territorial (P/T) surveillance systems. The assessment made a deliberate effort to account for jurisdictional variability in surveillance practices, with specific attention to geographical data coverage, integration of laboratory and epidemiological data, and the inclusion of health equity considerations.
3. Evaluates the availability of disaggregated data to identify gaps in capturing AMR impacts on marginalized populations.

### Data sources

The analysis was grounded in the 2025 AMR prioritization dataset, which applied a multi-criteria decision analysis (MCDA) framework using nine weighted criteria and Canadian data from 2017 to 2022. While the 2025 list was compared to the 2015 AMR prioritization in terms of pathogen inclusion and tier movement, the underlying data and respective sources used in the 2015 exercise were not re-analyzed [16].

Supplementary data sources included internal documentation and surveillance outputs from national and provincial/territorial (P/T) programs, such as: AMRNet [13], CNISP [14], the Canadian Integrated Program for Antimicrobial Resistance Surveillance (CIPARS) [17], the Antimicrobial Resistance and Nosocomial Infections (ARNI) program [18], ESAG [15], the Gonococcal Antimicrobial Surveillance Program (GASP) [19], the national laboratory surveillance system for invasive streptococcal disease (eSTREP) [20], and the Canadian Tuberculosis Laboratory Surveillance System (CBTLSS) [21]. P/T-specific surveillance reports were included where available [22–28].

Additional evidence was drawn from Canadian clinical guidelines, grey literature, peer-reviewed publications, and expert consultation with relevant PHAC program areas [29–40].

Majority of the data used for this analysis are surveillance datasets internal to the PHAC and its surveillance partners. Comprehensive details on data sources, extraction criteria, and synthesis processes are provided in Paper 1 of this series (“Canada’s 2025 AMR Priority Pathogens: Evidence-Based Ranking and Public Health Implications,” *Abdesselam, K. et al., 2025*) [12], including a full list of national and partner surveillance systems available in the supplementary documentation. The present analysis draws on these standardized national surveillance outputs and associated quality-assurance procedures to ensure consistency and comparability across datasets.

### Assessment approach

A qualitative assessment was conducted to evaluate how the 2025 AMR Priority Pathogen List aligns with Canada’s current AMR surveillance landscape and to identify areas where improvements may be warranted. The purpose was not to prescribe program-specific actions, but rather to explore the extent of alignment with existing systems, highlight opportunities for strengthening surveillance, and acknowledge ongoing fiscal and jurisdictional considerations that shape public health responses across Canada.

This assessment involved three complementary components:

1. **Prioritization review:**

A comparative analysis was performed to examine changes between the 2015 and 2025 prioritization exercises, including shifts in pathogen inclusion/exclusion, tier rankings, and methodological enhancements, particularly the integration of Canadian data and the introduction of a health equity criterion. In addition, the 2025 AMR Priority Pathogen List was compared to international prioritization frameworks developed by the World Health Organization (WHO) [41] and the U.S. Centers for Disease Control and Prevention (CDC) [42] to assess alignment, convergence, and areas of divergence.

1. **Surveillance System Mapping:**

Each of the 29 prioritized pathogens was mapped against PHAC’s current surveillance infrastructure. Surveillance coverage was categorized into three levels:

(1) enhanced national surveillance (integrated laboratory and epidemiologic data);
(2) routine national surveillance with limited standardization and/or provincial/territorial-level monitoring without national coordination via surveillance or research; and
(3) no formal surveillance;
(4) Equity and Data Granularity Analysis.

A targeted review was conducted to assess the availability and quality of disaggregated data related to geography, sociodemographic factors, and behavioural risk. This analysis aimed to determine the capacity of current systems to identify and address disproportionate AMR impacts on marginalized populations. Particular attention was given to recurring data limitations and the potential to incorporate equity-focused variables in future surveillance initiatives, where appropriate.

### Qualitative analysis and visualization

Qualitative analyses were conducted using Microsoft Excel [43] and R (v4.5.0) [44] to support the interpretation of surveillance alignment and equity-related findings. Visual outputs, including quadrant maps, summary tables, and system maturity diagrams, were developed using Microsoft 365 [43] tools, Visual Studio (Python) [45], and CANVA [46] to illustrate surveillance coverage, integration, and persistent data gaps.

## Results

### Prioritization Review

The 2025 AMR Priority Pathogen List identified 29 pathogens, each assigned to one of four tiers using a MCDA framework. This updated approach incorporates nine weighted criteria: incidence, trend, mode of transmission, case fatality ratio, morbidity, treatability, detection, health equity, and preventability. Compared to the 2015 prioritization, which identified 32 AMR pathogens but did not systematically assess them due to time constraints, the current exercise reflects significant methodological improvements [16]. In 2015, internal PHAC expertise and available Canadian surveillance data [16] and the CDC AMR Threat Report (2013) [42] were used as the primary basis for prioritization, and only 11 of the 32 pathogens included Canadian data, while the remainder relied on international sources. Further details on the full weighting scheme, data sources, and sensitivity analyses are available in the companion paper, Canada’s 2025 AMR Priority Pathogens: Evidence-Based Ranking and Public Health Implications (*Abdesselam, K. et al., 2025*) [12].

In contrast, the 2025 prioritization is based primarily on Canadian data from 2017–2022, with only a few criteria for two pathogens supplemented by international data due to domestic data gaps. The 10-year projection criterion was considered separately in the 2025 analysis through a scenario-based foresight initiative not covered in this paper. A health equity criterion was added for the first time to reflect PHAC’s broader commitment to addressing health disparities.

**Fig 1:**
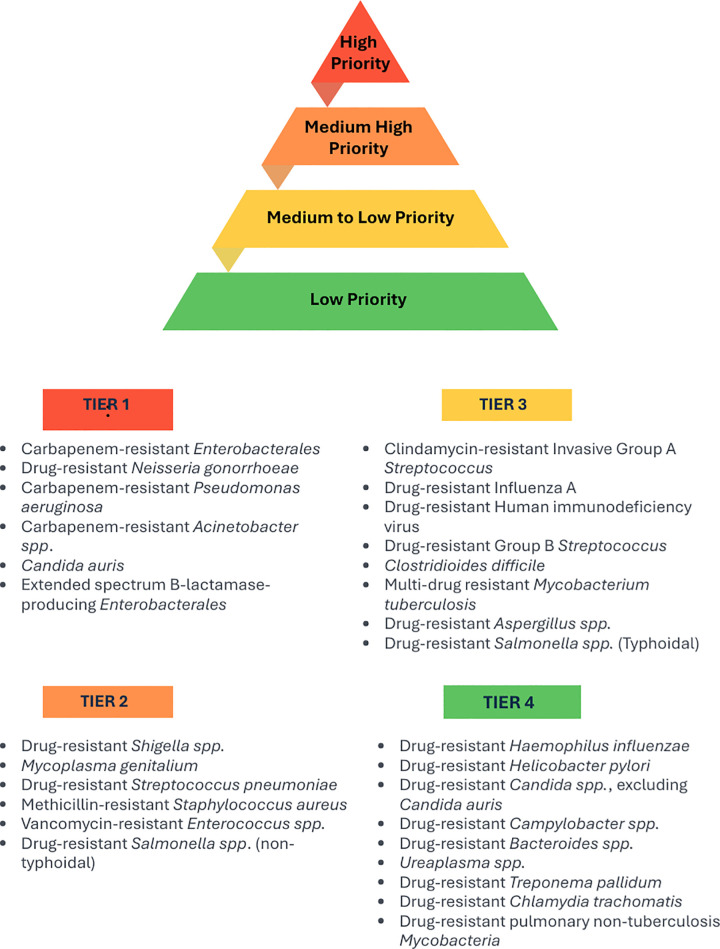
Canada’s Priority AMR Pathogens (2025), Grouped by Risk Tier Based on MCDA Evaluation.

These methodological refinements enabled a more accurate and contextually relevant assessment of AMR threats in Canada. As shown in Fig 1, the 29 assessed pathogens were stratified into tiers based on their relative public health threat and urgency of response. Tier 1 and Tier 2 pathogens, comprising 12 organisms, were designated as the highest priority for coordinated surveillance, resource allocation, and public health intervention due to their elevated resistance profiles, transmission dynamics, and disproportionate impacts on marginalized populations. However, pathogens not currently classified within Tier 1 or 2 should not be ignored or deprioritized. AMR is continuously evolving, and emerging resistance patterns may shift the threat landscape, requiring ongoing vigilance across all assessed organisms.

### Surveillance system mapping

**Fig 2:**
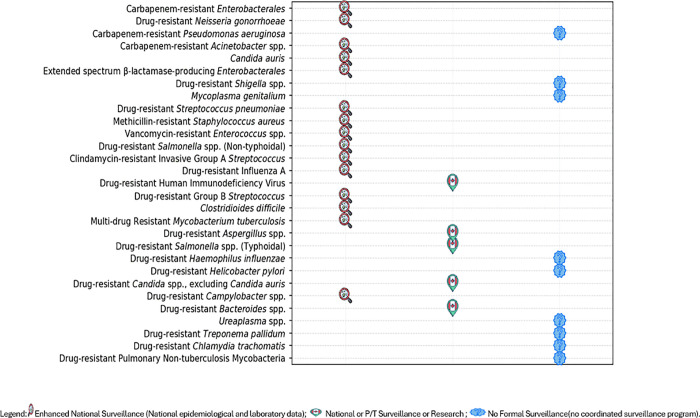
Surveillance Coverage of Priority AMR Pathogens across Canada by Level of Data Capture (Excludes AMRNET Surveillance Capacity) Depicts overall surveillance categories (enhanced, routine, or none) across Canadian jurisdictions, integrating information from AMRNet, CNISP, ESAG, CIPARS, and other national and P/T systems. ***Note****: CRE and CRA are classified under enhanced national surveillance. However, since 2016* [47], *CNISP transitioned from tracking CRE to CPE and from CRA to CPA. As a result, current epidemiological and laboratory data collected by CNISP reflect CPE and CPA specifically. Laboratory data on CRE and CRA continue to be collected by ARNI.* ***Note:*** *The surveillance coverage presented in this figure is based solely on publicly available information. This does not imply that surveillance or research activities are absent at the provincial or territorial level for certain pathogens; such efforts may exist but are not captured due to limited public disclosure*.

Among the 29 priority AMR pathogens identified in the 2025 assessment, 52% (15/29) are currently monitored through national enhanced surveillance systems, compared to fewer than 10 pathogens under coordinated national surveillance in 2015, defined as those that integrate both laboratory and epidemiological data (Fig 2). In parallel, AMRNet, a national laboratory-based surveillance platform, revealed that 90% (26/29) of identified pathogens have the potential to be systematically captured across participating jurisdictions (Table 1). AMRNet was launched in 2018 as a pilot in four provinces and territories.

**Table 1:** Laboratory-Based Surveillance Capacity for Monitoring AMR Priority Bacterial Pathogens Across Canadian Jurisdictions via AMRNet.

AMR Pathogen (n/N)	Provinces & Territories												
	AB	BC	MB	NB	NL	NS	NWT	NU	ON	PEI	QC	SK	YT
**Bacteria (23/24)**	✓	✓		✓	✓		✓		✓	✓		✓	
**Fungi (3/3)**	✓	✓		✓	✓		✓		✓	✓		✓	
**Viruses (0/2)**													
**Total**	90% (26/29)												

**Note:** AMRNet was first piloted in 2018 with participation from three provinces (Ontario, Saskatchewan, Prince Edward Island) and one territory (Northwest Territories). As of 2025, it has expanded to seven provinces and one territory, reflecting significant progress in national coordination. While the lab-based infrastructure is capable of capturing a wide range of bacterial AMR pathogens, current implementation remains limited: only four pathogens (approximately 15%) are consistently analyzed and publicly reported across all participating jurisdictions. This number is expected to increase as regions within provinces and territories are progressively onboard.

As of 2025, AMRNet includes participation from seven of ten provinces and one of three territories [48]. AMRNet collects antimicrobial susceptibility testing results from clinical isolates across all bacterial and fungal pathogens [13], with a database of over 50 million records. Despite the volume of data collected, public reporting as of 2025 has been limited to 13 pathogens. To date, all provinces, except for Manitoba and Quebec, and one territory (Northwest Territories) are participating, while Nunavut and Yukon have yet to join [48]. AMRNet does not capture viral AMR pathogens, such as drug-resistant HIV and Influenza A, and data beyond standard susceptibility testing remain limited. For example, ESBL status is included when available from incoming data streams, but AMRNet does not systematically capture ESBL information across all *Enterobacterales* [48]. Surveillance coverage remains strongest for bacterial and fungal pathogens, with Alberta, British Columbia, and Ontario demonstrating the most comprehensive and sustained reporting [14].

**Fig 3:**
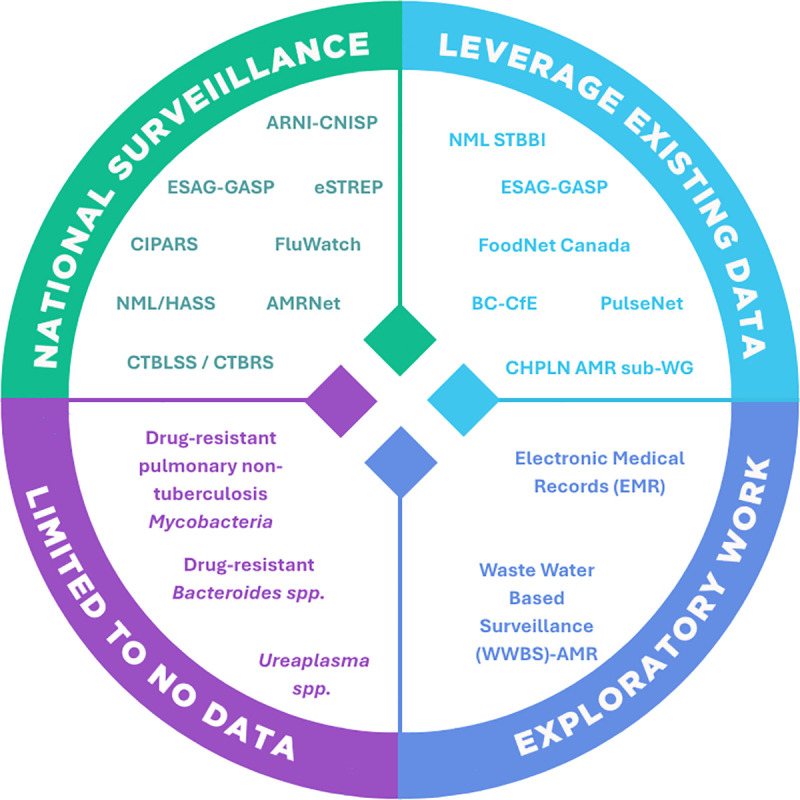
Mapping PHAC AMR surveillance and data sources to the 2025 Priority Pathogen List and the CARSS framework. Illustrates PHAC-specific program coverage and integration across four domains (National Surveillance, Leverage Existing Data, Exploratory Work, and Limited/No Data). *For detailed descriptions of surveillance sources and pathogen mapping, see Supplemental* *S1 Appendix*. ***Note:*** The BC Centre for Excellence in HIV/AIDS (BC-CfE) is the primary source for HIV drug resistance data in Canada, following the discontinuation of NMLB molecular HIV surveillance.

To further evaluate the breadth and duration of surveillance efforts across the prioritized pathogens, a quadrant-style framework was developed (Fig 3). This framework categorizes surveillance programs into four domains: National Surveillance, Leveraging Existing Data, Exploratory Work, and Limited to No Data. National Surveillance category includes systems currently monitored by PHAC, representing established national-level infrastructure. Leveraging existing data refers to P/T sources or networks in which PHAC is actively engaged, offering potential to enhance national surveillance through existing collaborations. Exploratory work encompasses initiatives led by PHAC to address known surveillance gaps and expand coverage. Limited to no data category captures pathogens or areas with minimal or no existing data sources at either the national or P/T level.

Well-established programs such as AMRNet [13,48], CNISP [14], and ESAG/GASP [15,19] support national-level surveillance for several pathogens, while other pathogens are partially captured through systems like FoodNet Canada [49], PulseNet Canada [50], and the Canadian Public Health Laboratory Network AMR sub-working group [51]. Exploratory efforts at the Public Health Agency of Canada, including the use of electronic medical records (EMR) in primary care settings, and wastewater-based surveillance (WWBS-AMR) [52,53] offer opportunities for expanded data collection. However, some gaps remain: *Ureaplasma spp***.** and drug-resistant *Bacteroides spp.* currently fall under the Limited to “No Data category”.

Comparison of the 2025 and 2015 AMR prioritization exercises revealed several important tier changes among pathogens previously identified as high priority (Table 2). Five pathogens demonstrated upward shifts in priority: drug-resistant *Neisseria gonorrhoeae*, carbapenem-resistant *Pseudomonas aeruginosa* (CRP), carbapenem-resistant *Acinetobacter spp.* (CRA), drug-resistant *Shigella spp.*, and drug-resistant *Streptococcus pneumoniae*, indicating rising resistance, clinical burden, and/or increased concern of community acquired infections. Notably, methicillin-resistant *Staphylococcus aureus* (MRSA) moved from Tier 1 to Tier 2. This reclassification reflects both a refinement in surveillance scope, expanding from bloodstream infections only (as assessed in 2015) to all MRSA infections, and potential improvements in infection prevention and control measures, especially within the healthcare setting [37,54]. Two pathogens, *Candida auris* and *Mycoplasma genitalium*, were newly prioritized in 2025, underscoring their recent emergence and growing clinical relevance in Canada. These shifts highlight the evolving nature of AMR threats and reinforce the need for regular reassessment to ensure prioritization frameworks remain responsive to current epidemiological realities.

**Table 2:** Comparison of 2025 Tier 1 & 2 to Their 2015 Assignments: Shifts in AMR Pathogen Tier Rankings (↑= moved up, ↓= moved down, ≡ = no change).

AMR Pathogens	2025 Tier	2015 Tier	Shift
Carbapenem-resistant *Enterobacterales*	1	1	≡
Drug-resistant *Neisseria gonorrhoeae*	1	2	↑
Carbapenem-resistant *Pseudomonas aeruginosa*	1	3	↑
Carbapenem-resistant *Acinetobacter* spp.	1	2	↑
*Candida auris*	1	–	N/A
Extended spectrum B-lactamase-producing *Enterobacterales*	1	1	≡
Drug-resistant *Shigella* spp.	2	4	↑
*Mycoplasma genitalium*	2	–	N/A
Drug-resistant *Streptococcus pneumoniae*	2	3	↑
Methicillin-Resistant *Staphylococcus aureus*	2	1	↓
Vancomycin-resistant Enterococcus spp.	2	2	≡
Drug-resistant *Salmonella* spp. (non-typhoidal)	2	3	↑

**Note:** In 2015, *multi-drug resistant (MDR) Acinetobacter spp.* and *MDR Pseudomonas aeruginosa* were assessed collectively, whereas in 2025, only *carbapenem-resistant* strains (CRA, CRPA) were included. The 2015 prioritization assessed only MRSA bloodstream infections (BSI), whereas the 2025 evaluation expanded to include all MRSA infections, leading to its tier shift. *Candida auris* and *Mycoplasma genitalium* were not assessed in 2015, reflecting their emergence as priority pathogens in the 2025 update.

Canada’s 2025 AMR Priority Pathogen List demonstrates strong alignment with international prioritization frameworks (Table 3). All Tier 1 pathogens and most Tier 2 pathogens are recognized in the WHO’s 2024 Bacterial Priority Pathogen List, the 2022 Fungal Priority Pathogen List, and the CDC’s 2019 AMR Threat Report [41,42,55]. For example, carbapenem-resistant *Enterobacterales*, *Acinetobacter spp.*, and *Candida auris* are all considered “Critical” by WHO and “Urgent” by the CDC, in alignment with Canada’s Tier 1 classifications. While *Mycoplasma genitalium* is not yet included in WHO’s lists, it is categorized in the CDC’s “Watch” tier, though this assessment is based on the 2019 report, and more recent data suggest increasing global concern due to rising macrolide and fluoroquinolone resistance [56,57]. Canada’s inclusion of this pathogen reflects emerging national evidence and highlights the value of timely updates to prioritization frameworks.

**Table 3:** Comparative Analysis of Canada’s Tier 1 and Tier 2 AMR Pathogens Against the WHO’s 2024 Bacterial Priority Pathogen List [41], and 2022 Fungal Priority Pathogen List [55], and the 2019 CDC Threat Report [42].

Priority Pathogen	PHAC	WHO	CDC
Carbapenem-resistant *Enterobacterales*	Tier 1	Critical	Urgent
Drug-resistant *Neisseria gonorrhoeae*	Tier 1	High	Urgent
Carbapenem-resistant *Pseudomonas aeruginosa*	Tier 1	High	Serious
Carbapenem-resistant *Acinetobacter* spp.	Tier 1	Critical	Urgent
*Candida auris*	Tier 1	Critical	Urgent
Extended spectrum B-lactamase-producing *Enterobacterales*	Tier 1	Critical	Serious
Drug-resistant *Shigella* spp.	Tier 2	High	Serious
*Mycoplasma genitalium*	Tier 2	N/A	Watch
Drug-resistant *Streptococcus pneumoniae*	Tier 2	Medium	Serious
Methicillin-Resistant *Staphylococcus aureus*	Tier 2	High	Serious
Vancomycin-resistant Enterococcus spp.	Tier 2	High	Serious
Drug-resistant *Salmonella* spp. (non-typhoidal)	Tier 2	High	Serious

**Note**: The WHO prioritization framework classifies pathogens from highest to lowest priority as Critical, High and Medium [41]. The US CDC classification uses Urgent, Serious and Concerning categories [42]. *Mycoplasma genitalium* is not currently included in the WHO’s 2024 Bacterial.

### Equity and data granularity analysis

**Fig 4:**
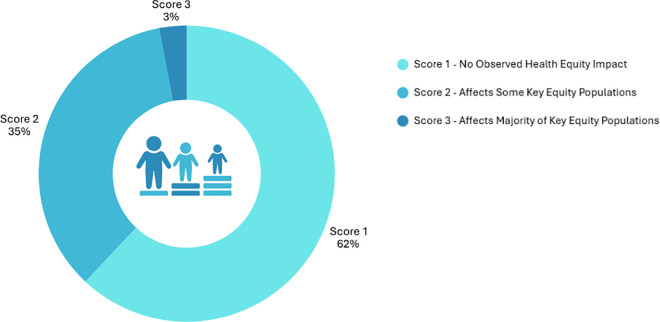
Distribution of priority AMR pathogens with identified equity concerns. Illustrates the distribution of Canada’s 2025 priority AMR pathogens according to their scores under the *health equity criterion* from the 2025 AMR prioritization exercise. **Score 1- No Observed Health Equity Impact:** No or minimal evidence of health equity impact. This includes either a lack of data or only limited, single-source information suggesting potential inequities or indirect indications of disproportionate burden. **Score 2 – Affects Some Key Equity Populations**: Evidence from multiple Canadian sources indicating disproportionate burden among one or more key equity populations. **Score 3 – Affects Majority of Key Equity Populations**: Strong or consistent evidence of inequitable burden across four of more of the key equity populations (e.g., Indigenous communities, gbMSM, PWID, unhoused populations, newcomers, sex workers) identified. *For detailed scoring descriptions and data sources, see Supplementary* *S2 Appendix* *(Equity-Oriented Surveillance Summary)*.

**Fig 5:**
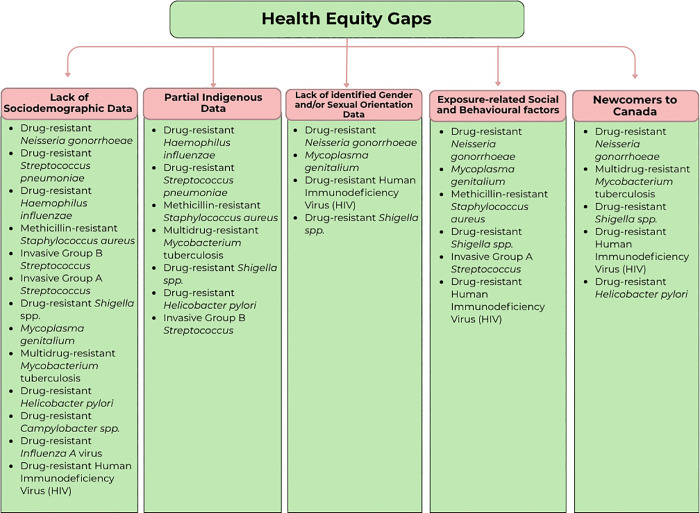
Qualitative review of health equity gaps among Canada’s 2025 priority AMR pathogens with documented equity considerations. *For additional information refer to* *S2 Appendix**. Equity-Oriented Surveillance Summary of Antimicrobial Resistance (AMR) Pathogens in Canada.* [31,56,57,58].

A key innovation in the 2025 framework was the integration of health equity as a formal prioritization criterion. Stratified analysis across the 29 pathogens (Figs 4 and 5) revealed that 35% of pathogens affected at least one key equity populations, and 3% were assessed to disproportionately impact the majority of these groups. These key equity populations included Indigenous communities, people who inject or use drugs (PWID), sex workers, gbMSM (gay, bisexual, and other men who have sex with men), unhoused populations, and newcomers including those from conflict- or disaster-affected regions [28,57–67]. For the remaining 62% of pathogens, no equity impact was identified based on current evidence [68]. However, this absence is potentially reflective of data gaps rather than of true equity neutrality. These percentages were derived from the 2025 prioritization exercise described in Paper 1 [12], wherein pathogens that received a score of 2 or 3 under the health equity criterion were classified as having documented or high-likelihood equity impacts.

A qualitative review of national surveillance systems and data collection processes was conducted to assess how equity considerations are currently integrated across priority AMR pathogens. This analysis, summarized in Fig 5, identified five recurring data limitations that hinder comprehensive equity assessment: (1) insufficient sociodemographic data; (2) limited Indigenous-specific data; (3) lack of gender and sexual orientation data; (4) inadequate characterization of exposure-related social and behavioural factors; and (5) underrepresentation of newcomers to Canada. These data gaps were especially prominent for pathogens such as *Neisseria gonorrhoeae* [62,63], *Mycoplasma genitalium* [31,56,57,58], multidrug-resistant *Mycobacterium tuberculosis* [21,69–71], and community-associated *MRSA* [54], constraining the ability to quantify disparities and develop targeted public health strategies. These findings underscore the need to strengthen equity-informed national surveillance and ensure consistent disaggregated data collection in future AMR monitoring efforts.

## Discussion

The 2025 AMR prioritization exercise marks a significant advancement in Canada’s national response to AMR, offering key insights to inform surveillance modernization and guide public health action. Since the 2015 iteration [16], notable progress has been made in data availability, methodological rigor, and the integration of health equity into prioritization frameworks. The Canadian Antimicrobial Resistance Surveillance System (CARSS) [9,10], launched by the Public Health Agency of Canada (PHAC) in 2015, has evolved from an integrative effort within the Agency to a broader coordination hub supporting policy, stewardship, and research.

Over the past decade, key milestones have included the implementation of AMRNet [13,48], now systematically collecting antimicrobial susceptibility laboratory data from inpatient and outpatient settings in 8 of the 10 provinces and 1 of the three territories in Canada, and the expansion of CNISP from 55 hospitals in 2015–109 hospitals across all 10 provinces and one territory by 2025 [14]. PHAC has also supported enhanced surveillance for emerging fungal threats like *Candida auris* [22,72], formalized an AMR-focused laboratory sub-working group within the Canadian Public Health Laboratory Network (CPHLN) [51], and piloted the use of electronic medical records and wastewater-based surveillance for community-level AMR gene trend detection. Despite these advancements, AMRNet participation remains uneven across jurisdictions, and resource constraints continue to limit broader data integration and analytic capacity. Still, these developments represent meaningful progress toward a more responsive and future-ready AMR surveillance system.

Carbapenem-resistant organisms (CROs), including CRE, CRP and CRA, were identified among Canada’s highest-priority AMR threats. These pathogens are especially concerning when they produce carbapenemases, which facilitate horizontal gene transfer of resistance traits [26,73]. While general carbapenem resistance is captured through AMRNet’s phenotypic susceptibility testing data, identifying carbapenemase-producing organisms (CPOs) requires molecular tools such as whole genome sequencing (WGS) [74]. CNISP [14] and ARNI [18] have been tracking hospital-acquired CPO-producing *Enterobacterales* and *Acinetobacter spp.* since 2017. However, there is no dedicated national enhanced surveillance system for carbapenemase-producing *Pseudomonas aeruginosa*, underscoring a critical surveillance gap.

The emergence of pathogens like *Candida auris* and *Mycoplasma genitalium* in the 2025 prioritization highlights the dynamic nature of the AMR landscape. *C. auris*, first detected in Canada in 2012 [22], remains underreported despite enhanced CNISP surveillance launched in 2019. Like most surveillance programs, surveillance of *C. auris* is currently voluntary and varies across provinces. Only British Columbia, Alberta, and Ontario have designated *C. auris* as a notifiable disease [22], while Quebec mandates immediate reporting through alternate mechanisms. Surveillance gaps may be exacerbated by challenges in identification, a high proportion of colonized cases, and disruptions caused by the COVID-19 pandemic [61]. Additionally, the lack of a dedicated national mycology reference laboratory limits Canada’s ability to perform consistent species-level identification, susceptibility testing, and detection of antifungal resistance mechanisms [75]. Only three fungal pathogen groups (*C. auris*, drug-resistant *Aspergillus spp.*, and other *Candida spp.*) were found to pose a risk in this exercise, suggesting that emerging fungal threats may remain under-characterized due to current limitations in surveillance and detection capacity [22,39,72].

Drug-resistant sexually transmitted infections (STIs) were also more prominently featured in the 2025 prioritization. *Neisseria gonorrhoeae* and *Shigella* spp. were ranked as Tier 1 and Tier 2 pathogens, respectively, due to rising resistance to first-line treatments and their disproportionate burden among key equity populations, including gay, bisexual, and other men who have sex with men (gbMSM) [59,63]. The integration of health equity as a formal prioritization criterion enabled a more nuanced evaluation of how AMR risks intersect with social and structural inequities, reinforcing the need for public health interventions that address both biological causes and other underlying determinants of health. Canada currently monitors AMR in *N. gonorrhoeae* through the Gonococcal Antimicrobial Surveillance Program (GASP) [15], which provides national-level laboratory-based resistance data. Additionally, the ESAG program, initially piloted in 2014 in four provinces and territories, has expanded to six P/Ts as of 2024, enabling enhanced epidemiological and behavioral data collection to better understand AMR risk in key equity populations [15].

*Shigella* spp. is a nationally notifiable disease and has historically been classified as a foodborne pathogen [24,27,28]. Its emerging role as a sexually transmitted infection, particularly among gbMSM, presents new public health challenges [28]. Despite its Tier 2 classification, Canada lacks a dedicated national AMR surveillance system for drug-resistant *Shigella spp.* [24]*.*AMR monitoring at the national level is currently limited to globally circulating ‘extensively drug resistant’ strains (XDR) of *Shigella* isolates (NMLB personal communication). To address this gap, PHAC, in collaboration with provincial and territorial (P/T) partners, is leveraging existing programs such as FoodNet Canada [49], a sentinel surveillance system monitoring enteric disease and exposures, and AMRNet [13,48] which collects lab-based AMR data are being leveraged to examine case data and resistance trends, providing a foundation for more comprehensive surveillance [24].

These partnerships are essential to understanding the burden of multidrug-resistant (MDR) and extensively drug-resistant (XDR) *Shigella spp.* and tailoring interventions accordingly.

The prioritization of *Mycoplasma genitalium* also reflects a growing public health concern. In 2024, 80% of *M. genitalium* isolates received and tested at the National Microbiology Laboratory (NML) showed macrolide resistance, a sharp increase from 51% in 2017. Surveillance for *M. genitalium* remains fragmented and largely dependent on voluntary sampling within broader STI surveillance systems [56]. Macrolide resistance has exceeded 50% in many settings [56], while quinolone resistance has been documented at rates as high as 32.7% in urban centers [58]. Findings from the Engage Cohort Study indicate even higher resistance among gbMSM (82%) [58], and data from Western Canada show that more than half of *M. genitalium* infections among Indigenous women are macrolide-resistant [58]. These disparities emphasize the urgent need for tailored and equitable AMR interventions. Although *M. genitalium* is not currently a nationally notifiable disease, designating it as such could help quantify its burden, as well as help emphasize its rising importance in the realm of AMR. However, notification alone would not ensure effective AMR monitoring. A more robust approach would involve integrating *M. genitalium* surveillance with laboratory-based platforms such as AMRNet and enhancing collaboration with equity-focused systems like ESAG, particularly given *M. genitalium*’s frequent co-infection with *N. gonorrhoeae* [76]. Strengthening national capacity to monitor resistance trends in this pathogen is critical to informing treatment guidelines and addressing emerging inequities in STI-related AMR.

Beyond *Mycoplasma genitalium*, emerging resistance has also been documented in *Chlamydia trachomatis* and *Treponema pallidum* [77]. Although current first-line treatments remain effective, ongoing surveillance is essential to detect shifts in resistance patterns early [77]. The synergistic nature of certain STIs, combined with the evolving adaptability of microorganisms and changing human behaviours, such as inconsistent use of barrier protection and decreased emphasis on safe sex practices, can accelerate resistance development [77]. These factors underscore the importance of maintaining robust surveillance to safeguard treatment efficacy and proactively adapt public health responses.

Until 2015, the National Microbiology Laboratory Branch (NMLB) led national molecular surveillance of HIV drug resistance in Canada [78]. Since its discontinuation, this responsibility has not been formally transferred to another federal public health entity. The BC Centre for Excellence in HIV/AIDS (BC-CfE) [79], Canada’s largest HIV research, treatment, and education facility, now plays a central role in coordinating HIV drug resistance research and reporting, in collaboration with provincial and territorial partners and in support of Canada’s international commitments. However, growing concerns have emerged around the rise of drug-resistant HIV globally and in Canada, particularly in the context of immigration and refugee health, where access to early testing and appropriate treatment may be variable [80]. To address this evolving threat, there is a need for renewed national coordination and investment in HIV drug resistance surveillance. There may be an opportunity for PHAC to support BC-CfE in exploring options for a sustainable, nationally integrated system to detect, monitor, and respond to HIV resistance trends across jurisdictions.

The 2025 prioritization found that 28% of assessed AMR pathogens exhibit resistance to at least one recommended first-line treatment in Canada [12], based on Canadian clinical guidelines and, where necessary, syndromic approaches [28–38,69,77].While this treatment criterion offered a standardized benchmark, it also exposed critical gaps in available data. For carbapenem-resistant organisms (CROs), AMRNet provides some epidemiological data from both healthcare and community settings, but this is not comprehensive and does not include molecular testing to determine specific resistance mechanisms. Enhanced epidemiological and laboratory data for CPE and CPA are available through CNISP but are limited to hospital settings. No routine molecular data is collected for CPP or for the broader CPO group across all settings. These surveillance limitations hinder the ability to fully characterize resistance mechanisms, assess treatment implications, and capture the breadth of transmission across settings. Expanding molecular surveillance to capture enzyme-type data for all CPOs, particularly beyond acute care, would address these gaps, strengthen treatment assessments, and improve the robustness of future prioritization exercises.

The reclassification of MRSA from Tier 1 in 2015 to Tier 2 in 2025 reflects both methodological changes and evolving epidemiology. The 2025 assessment included all MRSA infections, compared to the 2015 focus on bloodstream infections only [37,54]. Strengthened infection prevention and control (IPC) measures and expanded surveillance have likely contributed to declining rates for healthcare-associated MRSA [81]. However, CNISP data from 2018–2022 show a shift in MRSA-BSIs toward community acquisition [10], highlighting the need to strengthen surveillance and IPC strategies outside hospital settings, particularly in populations disproportionately affected by systemic barriers, such as people who use drugs or unhoused individuals.

Drug-resistant Influenza A, ranked as a Tier 3 priority, remains a public health concern due to its potential to trigger a pandemic [82]. Although currently less pressing than many bacterial or fungal threats, the emergence of resistance to neuraminidase inhibitors, particularly oseltamivir, could markedly undermine treatment effectiveness, given the narrow therapeutic window for influenza [82]. PHAC addresses this risk through FluWatch, Canada’s year-round influenza surveillance program, which monitors geographic spread, severity, laboratory detections, antiviral resistance, vaccine effectiveness, and outbreak activity [83]. The NMLB routinely tests influenza isolates for antiviral resistance and works closely with provincial and territorial laboratories to identify novel strains [83]. Current clinical guidance recommends early antiviral treatment for high-risk groups, with zanamivir as an alternative to oseltamivir; amantadine is no longer recommended due to widespread resistance. By combining real-time reporting with ongoing strain characterization, these measures enable early detection of resistant strains and rapid public health response. Continued vigilance is essential, given influenza’s capacity for rapid evolution and global spread.

Notably, Multidrug-resistant tuberculosis (MDR-TB) did not rank among the highest-tier threats in the 2025 prioritization but remains a serious global and domestic health concern. Worldwide, MDR-TB incidence is being fueled by reduced international funding for TB control, ongoing conflicts, and disruptions to health systems [68]. In Canada, risk is heightened by immigration and tourism from high-incidence regions, including refugees and asylum seekers, international students, and temporary foreign workers. The disease disproportionately affects marginalized populations, particularly Indigenous communities and newcomers from TB endemic regions [20,67,68]. In Canada, treatment of MDR-TB is further complicated by persistent challenges related to drug accessibility and ongoing shortages, which undermine timely and effective care and exacerbate health inequities among affected populations [67,68].

Nationally, PHAC coordinates case-based surveillance through the Canadian Tuberculosis Reporting System (CTBRS) and provides confirmatory drug susceptibility testing via the NMLB [21]. Collaboration with provincial and territorial TB programs supports timely diagnosis, contact tracing, and treatment, in line with the Canadian Tuberculosis Standards, which incorporate newer agents such as bedaquiline [21,69–71]. Current efforts also focus on rapid diagnostics, provider training, and community outreach to reduce stigma and improve treatment adherence.

These integrated measures have supported Canada’s continued low incidence of MDR-TB [21], particularly through coordinated surveillance, timely diagnosis, and targeted interventions for vulnerable groups. However, sustained investment in rapid diagnostics, stronger surveillance integration, equitable access to second line and novel TB drugs, and tailored interventions will be critical to prevent MDR-TB from emerging as a larger domestic threat.

The 2025 prioritization aligns closely with global AMR classifications from WHO [41] and the CDC [42], reinforcing Canada’s commitment to international AMR mitigation while adapting to national contexts. Shared global concerns, such as carbapenem-resistant *Enterobacterales, C. auris*, and drug-resistant *N.* *gonorrhoeae*, are reflected in Canada’s Tier 1 priorities, alongside emerging threats informed by Canadian data and equity considerations.

Although AMRNet has the potential to capture resistance data for approximately 90% of prioritized pathogens, significant capacity gaps remain that limit its comprehensive surveillance coverage. Currently, seven provinces and one territory participate in AMRNet [13,48], and not all jurisdictions can report comprehensively across all organisms, specimen types or regions. A phased approach is underway to expand participation and build capacity, however expansion is currently paused in light of resource constraints. While AMRNet captures data on all tested bacterial/fungal pathogens, only a small number of pathogens are currently included in analyses and reporting. AMRNet also does not cover viral AMR pathogens and lacks routine genotypic testing, limiting insight into resistance mechanisms [13,48].

To build a comprehensive national surveillance strategy, AMRNet should complement, not replace, existing P/T and national program-specific systems. A multi-faceted approach that integrates laboratory, clinical, and sociodemographic data will be essential to capturing resistance trends across populations and settings. Investing in genomic and molecular surveillance, and supporting infrastructure across jurisdictions, will further enable timely detection and response.

Embedding health equity into AMR surveillance remains an ongoing challenge. Most current systems do not routinely collect disaggregated data on race, ethnicity, gender identity, housing status, or other key social determinants of health, limiting the ability to assess how AMR disproportionately affects marginalized populations [28,58]. The integration of health equity into the 2025 prioritization exercise was an important step forward, but we acknowledge that its treatment in this paper is primarily descriptive. Advancing beyond description will require operational frameworks embedded within surveillance programs themselves, recognizing that the federal role is to coordinate rather than prescribe. Nonetheless, this work surfaced several considerations that program areas may wish to explore, where feasible within existing surveillance infrastructure and where not already underway, including the use of standardized equity indicators, consistent methods for data disaggregation through linkage with administrative or laboratory datasets, and governance models that uphold Indigenous data sovereignty and support co-design with equity-deserving communities.

Work is already underway at the Public Health Agency of Canada to address these gaps, in alignment with the Chief Public Health Officer’s 2022–2025 Vision, which positions health equity as a central lens for public health action. This includes efforts to strengthen inclusion of underrepresented voices, support Indigenous self-determination in health, and improve governance through multi-sectoral collaboration. Programs such as ESAG offer partial models by integrating laboratory resistance testing with behavioural and demographic data collection, enabling equity-informed insights not available through traditional laboratory systems alone. Further integration of equity variables into existing AMR surveillance platforms, alongside complementary data sources such as EMRs and health administrative datasets, could help ensure that surveillance captures not only biological threats but also the structural and social contexts that shape vulnerability. Importantly, such data must be collected thoughtfully, with surveillance approaches that prioritize trust-building, privacy, and co-design with affected communities to ensure that equity-informed data collection is respectful, meaningful, and actionable.

## Conclusion

The findings of this study highlight the evolving landscape of AMR threats in Canada, underscoring the need for strengthened surveillance, early detection of emerging pathogens, and the systematic integration of health equity into national public health priorities. Since 2015, Canada has made substantial strides in surveillance capacity and coordination, supported by platforms such as AMRNet, CNISP, ESAG and targeted initiatives addressing fungal and sexually transmitted AMR threats.

Enhanced efforts are underway to monitor emerging pathogens such as *Candida auris*, *Mycoplasma genitalium*, and drug-resistant *Shigella spp.*, alongside expanded resistance tracking for *Neisseria gonorrhoeae* through GASP and ESAG. While these initiatives represent significant progress, gaps remain in molecular surveillance, equity-informed data collection, and real-time integration of laboratory and clinical information. AMRNet, in particular, offers a robust foundation for national AMR surveillance, and has the potential to capture resistance trends for 90% of prioritized pathogens. With sustained investment and broader jurisdictional participation, it can serve as a critical decision-support tool for public health authorities and antimicrobial stewardship programs. However, effective surveillance must extend beyond laboratory-confirmed cases to reflect population-specific risks, treatment outcomes, and transmission dynamics.

The absence of an acute public health emergency should not be interpreted as the absence of risk. Often, it is the result of years of steady, underrecognized public health effort. During times of fiscal constraint, as Canada and many other countries face budgetary pressures, it is especially important to preserve investments in public health infrastructure, including AMR surveillance. When funding is deprioritized in the absence of crisis, the long-term ability to prevent, detect, and respond to emerging threats is compromised.

Ultimately, Canada’s 2025 AMR prioritization framework offers critical insights to guide the path forward, enabling alignment with global surveillance priorities, strengthening preparedness for emerging and re-emerging threats, and embedding equity at the core of public health protection. Sustained collaboration with provincial and territorial partners, broader surveillance coverage, and stable, long-term investment will be essential to maintaining momentum. Translating these findings into action will not only bolster Canada’s current AMR response but also ensure resilience and equity in addressing future risks.

## Supporting information

S1 Appendix Fig 5. Mapping Existing AMR Surveillance Sources to the 2025 Priority Pathogen List and CARSS Framework Legend. (DOCX)

S2 Appendix Equity-Oriented Surveillance Summary of Antimicrobial Resistance (AMR) Pathogens in Canada [High-Priority AMR Pathogens (Score 2 or 3)]. (DOCX)

## Abbreviation:

AMR: Antimicrobial resistance
AMU: Antimicrobial use
AMRNet: Antimicrobial Resistance Network (national laboratory-based AMR surveillance platform)
ARNI: Antimicrobial Resistance and Nosocomial Infections (program)
BC-CfE: BC Centre for Excellence in HIV/AIDS
CARSS: Canadian Antimicrobial Resistance Surveillance System
CBTLSS: Canadian Tuberculosis Laboratory Surveillance System
CDC: U.S. Centers for Disease Control and Prevention
CIPARS: Canadian Integrated Program for Antimicrobial Resistance Surveillance
CNISP: Canadian Nosocomial Infection Surveillance Program
CPHLN: Canadian Public Health Laboratory Network
CPE: Carbapenemase-producing Enterobacterales
CPO(s): Carbapenemase-producing organisms
CRA: Carbapenem-resistant Acinetobacter spp.
CRE: Carbapenem-resistant Enterobacterales
CRP/CRPA: Carbapenem-resistant Pseudomonas aeruginosa (CRPA is species-explicit)
CRO(s): Carbapenem-resistant organisms
CTBRS: Canadian Tuberculosis Reporting System
EMR(s): Electronic medical record(s)
ESAG: Enhanced Surveillance of Antimicrobial Resistant Gonorrhea
ESBL: Extended-spectrum β-lactamase
FluWatch: Canada’s National Influenza Surveillance Program
GASP: Gonococcal Antimicrobial Surveillance Program
GHSA: Global Health Security Agenda
HIV: Human immunodeficiency virus
IPC: Infection prevention and control
MCDA: multi-criteria decision analysis
MDR: Multidrug-resistant
MRSA: Methicillin-resistant Staphylococcus aureus
NML: National Microbiology Laboratory
NMLB: National Microbiology Laboratory Branch
PCAP: Pan-Canadian Action Plan on AMR
PHAC: Public Health Agency of Canada
PWID: People who inject/use drugs
SDGs: United Nations Sustainable Development Goals
STI(s): Sexually transmitted infection(s)
TB: Tuberculosis
UN: United Nations
WHO: World Health Organization
WGS: Whole-genome sequencing
WWBS-AMR: Wastewater-based surveillance for AMR
gbMSM: Gay, bisexual, and other men who have sex with men
XDR: Extensively drug-resistant
AB: Alberta
BC: British Columbia
MB: Manitoba
NB: New Brunswick
NL: Newfoundland and Labrador
NS: Nova Scotia
NT: Northwest Territories
NU: Nunavut
ON: Ontario
PE: Prince Edward Island
QC: Quebec
SK: Saskatchewan
YT: Yukon
