# Supplementary material for: AMR surveillance in Canada: Insights from the 2025 Priority Pathogen List
Source: PLoS One. 2026 Feb 18;21(2):e0341133. doi: 10.1371/journal.pone.0341133 (PMC12915920; doi:10.1371/journal.pone.0341133)
Supplement: S1 Appendix — (DOCX) [file pone.0341133.s001.docx]

**S1 Appendix. Figure 5. Mapping Existing AMR Surveillance Sources to the 2025 Priority Pathogen List and CARSS Framework Legend**

| Surveillance Category | Surveillance Source | Pathogen |
| --- | --- | --- |
| National Surveillance | The Antimicrobial Resistance and Nosocomial Infections (**ARNI**) laboratory - The Canadian Nosocomial Infection Surveillance Program (**CNISP**) | Carbapenem-resistant *Enterobacterales* |
|  |  | Carbapenem-resistant *Acinetobacter spp.* |
|  |  | *Candida auris* |
|  |  | Methicillin-resistant *Staphylococcus aureus* (MRSA) |
|  |  | Vancomycin-resistant *Enterococcus spp*. (VRE) |
|  |  | *Clostridioides difficile* |
|  |  | Extended spectrum Beta-lactamase-producing *Enterobacterales* (ESBL) |
|  | The Enhanced Surveillance of Antimicrobial Resistant Gonorrhea (**ESAG**) program - The Gonococcal Antimicrobial Surveillance Program (**GASP**) | Drug-resistant *Neisseria gonorrhoeae* |
|  | Surveillance of Invasive Streptococcal Disease (**eSTREP**) | Drug-resistant *Streptococcus pneumoniae* |
|  |  | Drug-resistant Group B *Streptococcus* |
|  |  | Clindamycin-resistant Invasive Group A *Streptococcus* |
|  | Canadian Integrated Program for Antimicrobial Resistance Surveillance (**CIPARS**) | Drug-resistant *Salmonella spp.* (non-typhoidal) |
|  |  | Drug-resistant *Salmonella spp.* (Typhoidal) |
|  |  | Drug-resistant *Campylobacter spp.* |
|  |  | Extended spectrum Beta-lactamase-producing *Enterobacterales* (ESBL) |
|  |  |  |
|  | **FluWatch** (Agency Internal) | Drug-resistant Influenza A |
|  | **NML**/National HIV/AIDS Surveillance System (**HASS**) | Human Immunodeficiency Virus (HIV) |
|  | **AMRNet** | *Salmonella Typhi* |
|  |  | Invasive non-typhoidal *Salmonella* |
|  |  | *Klebsiella pneumoniae* |
|  |  | *Neisseria Gonorrhoeae* |
|  |  | *Escherichia coli* |
|  |  | Diarrheagenic *E. coli* |
|  |  | *Shigella and Enteroinvasive Escherichia coli (EIEC)* |
|  | The Canadian Tuberculosis Laboratory Surveillance System (**CTBLSS**) / The Canadian Tuberculosis Reporting System (**CTBRS**) | Multi-drug-resistant *Mycobacterium tuberculosis* (MDR-TB) |
| Leveraging Existing Sources | National Microbiology Laboratory's (**NML**) - Sexually Transmitted and Blood-borne Infections (**STBBI**) | Sexually Transmitted Bloodborne Infections (STBBI) |
|  | **ESAG**-**GASP** | *Mycoplasma Genitalium* |
|  | **FoodNet**-**PulseNet** | Drug-resistant *Salmonella spp*. (non-typhoidal) |
|  |  | Drug-resistant *Salmonella spp*. (Typhoidal) |
|  |  | Drug-resistant *Campylobacter spp.* |
|  | The Canadian Public Health Laboratory Network (**CPHLN**) AMR sub-Working Group | *Pseudomonas aeruginosa* |
|  | BC Centre for Excellence in HIV/AIDS **(BC-CfE)** | Human Immunodeficiency Virus (HIV) |
| Exploratory Surveillance | **(WWBS)-AMR** |  |
|  | **EMR** (The Canadian Primary Care Sentinel Surveillance Network (**CPCSSN**)) |  |
| Limited Data Sources Available |  | *Drug-resistant Bacteroides spp.* |
|  |  | Drug-resistant pulmonary non-tuberculosis *Mycobacteria* |
|  |  | *Ureaplasma spp.* |
