# Supplementary material for: AMR surveillance in Canada: Insights from the 2025 Priority Pathogen List
Source: PLoS One. 2026 Feb 18;21(2):e0341133. doi: 10.1371/journal.pone.0341133 (PMC12915920; doi:10.1371/journal.pone.0341133)
Supplement: S2 Appendix — (DOCX) [file pone.0341133.s002.docx]

**S2. Equity-Oriented Surveillance Summary of Antimicrobial Resistance (AMR) Pathogens in Canada [High-Priority AMR Pathogens (Score 2 or 3)]**

| Pathogen | National Surveillance & Equity Indicators | Provincial/Territorial Systems | Populations Identified | Equity Gaps | Key References |
| --- | --- | --- | --- | --- | --- |
| Drug-resistant Neisseria gonorrhoeae (DR-NG) | ESAG (Enhanced Surveillance of Antimicrobial-Resistant Gonorrhea), GASP (Gonococcal Antimicrobial Surveillance Program), NML – limited equity indicators | Ontario collects limited sex/gender/sexual orientation; housing status not captured | Gay, bisexual, and other men who have sex with men (gbMSM); unhoused populations; youth and racialized individuals; travelers/newcomers | No routine collection of race, ethnicity, sexual orientation, housing, or immigration status; NAAT-based diagnoses limit resistance data | Sawatzky 2022; Lefebvre 2018; PHAC–GASP, ESAG Reports |
| Drug-resistant Invasive Group A Streptococcus (iGAS) | CNDSS, NML – no equity fields | Alberta, other provinces vary; outbreak-based | Children <15; older adults; unhoused and substance-using populations; Indigenous communities; postpartum individuals | Equity variables not reported in routine data; underrepresentation of small/remote jurisdictions | Golden 2024; Canadian Paediatric Society 2019 |
| Drug-resistant Streptococcus pneumoniae (DR-SPN) | CARSS, CANWARD, eSTREP, NML – no equity indicators | Provincial reporting inconsistent | Children <5; older adults; Indigenous and unhoused populations; substance users; low-income urban groups | No routine linkage with social determinants; vaccine coverage inequities not tracked | Golden 2015; Griffith 2024; PHAC–CARSS 2024 |
| Multi-drug Resistant Tuberculosis (MDR-TB) | CTBRS, PHAC TB Program; Indigenous Services Canada reports (limited public equity data) | Variable by province (BC, AB more detailed) | Indigenous Peoples (Inuit, FN, Métis); immigrants/newcomers; PLWH; unhoused; correctional populations | No routine sociodemographic data; fragmented Indigenous data governance; stigma barriers | PHAC 2022; Canadian TB Standards 2022; ITK 2018 |
| Drug-resistant Shigella spp. (DR-Shigella) | CIPARS (human module lacks equity); NML | Ontario PHO LIMS includes MSM/housing; limited elsewhere | MSM; unhoused; travelers (esp. Pakistan-linked) | No national-level data; marginalized populations underrepresented; limited Indigenous data | Stefanovic 2023; PHO 2023; NCCID 2024 |
| Drug-resistant Haemophilus influenzae (DR-HI) | PHAC NNDSS (post-2018, limited fields); CIRN; NML | PHOL (Ontario, detailed serotyping/AST) | Infants; older adults; Indigenous communities (esp. Hia); immunocompromised; LTC residents | Equity markers absent; historical underreporting of non-Hib strains; limited geographic data | McTaggart 2021; Desai 2015; PHAC 2023 |
| Drug-resistant Helicobacter pylori (DR-HP) | No national surveillance | Not collected | First Nations communities (high prevalence); older adults; Northeastern Ontario residents | No national system; fragmented, small-scale studies; no linkage with sociodemographic factors | Eng 2015; Aklavik H. pylori Project |
| Methicillin-Resistant Staphylococcus aureus (MRSA) | CNISP, NML – hospital-based, no equity fields | Community data sparse | People who inject drugs (PWID); incarcerated; Indigenous populations; hospitalized and dialysis patients; newborns | Equity markers absent; community-associated MRSA underrepresented; rural/remote gaps | Conly 2021; Conly & Johnston 2019; CNISP 2025 |
| Drug-resistant Invasive Group B Streptococcus (iGBS) | NML (limited); CNISP potential | Alberta, Ontario provincial surveillance | Neonates; pregnant people; postpartum infections | No race, income, immigration, or rural/urban breakdowns; no equity-linked maternal/neonatal outcomes | Alhhazmi 2016; Teatero 2015; PHAC/NML 2020 |
| Drug-resistant Campylobacter spp. | CIPARS (animal + human, limited equity) | SK historical lab data | Children; rural dwellers; immunocompromised; food-handlers/agricultural workers | No sociodemographic data; no clinical linkage; underpowered provincial reporting | PHAC–CIPARS 2022; Otto 2020 |
| Drug-resistant Mycoplasma genitalium (Mgen) | No national system; PHAC STI Guidelines | Montreal Engage study | gbMSM; people with persistent STI symptoms; urban clinic users | No routine testing or surveillance; limited access in rural/underserved groups | Lê 2023; PHAC 2021; CATIE 2023 |
| Drug-resistant Influenza A | FluWatch, NML, PHAC respiratory surveillance – no equity indicators | Regional vaccine uptake tracked, not AMR-focused | Adults 65+; children <5; pregnant/LTC residents; Indigenous and rural communities (barriers to care); poultry workers | No sociodemographic disaggregation; zoonotic risk lacks occupational/social markers | PHAC FluWatch 2025; PHAC Pathogen Safety Data Sheet 2023 |
| Drug-resistant HIV | NML genotyping & NGS; ICES–PHO (Ontario) | Ontario collects some sex/region equity data | Newly diagnosed PLWH; individuals with transmitted drug resistance; Indigenous, rural, low-income populations with barriers | Limited access to pre-treatment testing for women, Indigenous, rural populations; stigma; lack of national sociodemographic registry | Mbuagbaw 2021; CATIE 2024; PHAC–NML 2023 |

**References**

1. Sawatzky P, et al. 2022. Increasing Azithromycin Resistance in *Neisseria gonorrhoeae* Due to NG-MAST 12302 Clonal Spread in Canada, 2015–2018. *Antimicrob Agents Chemother* 66(3):e01688-21.
2. Lefebvre B, et al. 2018. Ceftriaxone-Resistant *Neisseria gonorrhoeae*, Canada, 2017. *Emerg Infect Dis* 24(2):381–383.
3. Public Health Agency of Canada (PHAC). 2014–2021. National Surveillance of Antimicrobial Susceptibilities of *Neisseria gonorrhoeae*, Annual Summary Reports.
4. Golden AR, et al. 2024. Invasive group A streptococcal disease surveillance in Canada, 2021–2022. *Can Commun Dis Rep* 50(5):121–134.
5. Canadian Paediatric Society. 2019, reaffirmed 2024. Invasive group A streptococcal disease: Management and chemoprophylaxis. *Paediatr Child Health* 24(2):128.
6. Golden AR, et al. 2015. Characterization of MDR and XDR *Streptococcus pneumoniae* in Canada, 2007–13. *J Antimicrob Chemother* 70(4):1185–1191.
7. Griffith A, Golden AR, Lefebvre B, et al. 2024. Invasive pneumococcal disease surveillance in Canada, 2021–2022. *Can Commun Dis Rep* 50(5):121–134.
8. Public Health Agency of Canada. 2024. Canadian Antimicrobial Resistance Surveillance System (CARSS) 2024 Report.
9. Public Health Agency of Canada. 2021. National Laboratory Surveillance of Invasive Streptococcal Disease in Canada – Annual Summary 2019.
10. Public Health Agency of Canada. 2022. Tuberculosis in Canada 2020 – Surveillance Report.
11. Canadian Thoracic Society, Menzies D, et al. 2022. *Canadian Tuberculosis Standards*, 8th Edition. Ottawa: Public Health Agency of Canada.
12. Inuit Tapiriit Kanatami (ITK). 2018. Inuit Tuberculosis Elimination Framework.
13. Stefanovic A, et al. 2023. Multidrug-Resistant *Shigella sonnei* Bacteremia among Persons Experiencing Homelessness, Vancouver, British Columbia, Canada. *Emerg Infect Dis* 29(8):1668–1671.
14. Public Health Ontario. 2023. *Shigella Antimicrobial Resistance*. Toronto: PHO.
15. National Collaborating Centre for Infectious Diseases (NCCID). 2024. *Shigellosis*.
16. McTaggart LR, et al. 2021. Increased Incidence of Invasive *Haemophilus influenzae* Disease Driven by Non-Type B Isolates in Ontario, Canada, 2014–2018. *Microbiol Spectr* 9(5):e00803-21.
17. Desai S, et al. 2015. The epidemiology of invasive *Haemophilus influenzae* non-serotype B disease in Ontario, 2004–2013. *PLoS One* 10(11):e0142179.
18. Public Health Agency of Canada. 2023. Pathogen Safety Data Sheet: *Haemophilus influenzae*.
19. Eng NF, et al. 2015. Antimicrobial susceptibility of Canadian isolates of *Helicobacter pylori* in Northeastern Ontario. *Can J Infect Dis Med Microbiol* 26(3):137–144.
20. Conly J, et al. 2021. National surveillance of MRSA bloodstream infections in Canada: Trends and implications. *J Antimicrob Chemother* 76(12):3086–3094.
21. Conly J, Johnston B. 2019. Community-associated MRSA in Canada: A review. *Can Commun Dis Rep* 45(1):5–10.
22. Public Health Agency of Canada. 2025. Canadian Nosocomial Infection Surveillance Program (CNISP) MRSA Surveillance Protocol.
23. Alhhazmi A, et al. 2016. Epidemiology and antimicrobial susceptibility of invasive group B streptococcus in Alberta, Canada. *Can J Infect Dis Med Microbiol* 2016:1467063.
24. Teatero S, et al. 2015. Emergence of serotype IV invasive group B streptococcus disease in Toronto, Canada. *Emerg Infect Dis* 21(4):585–591.
25. Public Health Agency of Canada/National Microbiology Laboratory. 2020. National Laboratory Surveillance of Invasive Streptococcal Disease in Canada.
26. Public Health Agency of Canada. 2024. Canadian Integrated Program for Antimicrobial Resistance Surveillance (CIPARS) 2022 Executive Summary.
27. Otto SJ, et al. 2020. Antimicrobial Resistance of Human *Campylobacter* Species in Saskatchewan, 1999–2006. *Foodborne Pathog Dis* 17(3):178–186.
28. Lê M, et al. 2023. *Mycoplasma genitalium* infection among gbMSM in Montreal, Canada. *Can Commun Dis Rep* 49(11/12):477–486.
29. Public Health Agency of Canada. 2021. Canadian Guidelines on Sexually Transmitted Infections – *Mycoplasma genitalium* Clinical Guidance.
30. CATIE. 2023. Researchers study *Mycoplasma genitalium* in Montreal. Toronto: CATIE.
31. Public Health Agency of Canada. 2025. FluWatch Reports (2023–2025).
32. Public Health Agency of Canada. 2023. Pathogen Safety Data Sheet: Influenza A (H5, H7, H9).
33. Public Health Agency of Canada. 2025. Respiratory Virus Surveillance Weekly Reports.
34. Mbuagbaw L, et al. 2021. Impact of routine HIV drug resistance testing in Ontario: A controlled interrupted time series study. *PLoS One* 16(9):e0257183.
35. CATIE. 2024. Drug Resistance and Resistance Testing. Toronto: CATIE.
36. Public Health Agency of Canada/National Microbiology Laboratory. 2023. HIV Drug Resistance Surveillance Reports.
